# Detection of axonal synapses in 3D two-photon images

**DOI:** 10.1371/journal.pone.0183309

**Published:** 2017-09-05

**Authors:** Cher Bass, Pyry Helkkula, Vincenzo De Paola, Claudia Clopath, Anil Anthony Bharath

**Affiliations:** 1 Centre for Neurotechnology, South Kensington Campus, Imperial College London, London, United Kingdom; 2 Department of Bioengineering, South Kensington Campus, Imperial College London, London, United Kingdom; 3 MRC Clinical Science Centre, Faculty of Medicine, Hammersmith Campus, Imperial College London, London, United Kingdom; National Institutes of Health, UNITED STATES

## Abstract

Studies of structural plasticity in the brain often require the detection and analysis of axonal synapses (boutons). To date, bouton detection has been largely manual or semi-automated, relying on a step that traces the axons before detection the boutons. If tracing the axon fails, the accuracy of bouton detection is compromised. In this paper, we propose a new algorithm that does not require tracing the axon to detect axonal boutons in 3D two-photon images taken from the mouse cortex. To find the most appropriate techniques for this task, we compared several well-known algorithms for interest point detection and feature descriptor generation. The final algorithm proposed has the following main steps: (1) a Laplacian of Gaussian (LoG) based feature enhancement module to accentuate the appearance of boutons; (2) a Speeded Up Robust Features (SURF) interest point detector to find candidate locations for feature extraction; (3) non-maximum suppression to eliminate candidates that were detected more than once in the same local region; (4) generation of feature descriptors based on Gabor filters; (5) a Support Vector Machine (SVM) classifier, trained on features from labelled data, and was used to distinguish between bouton and non-bouton candidates. We found that our method achieved a Recall of 95%, Precision of 76%, and F1 score of 84% within a new dataset that we make available for accessing bouton detection. On average, Recall and F1 score were significantly better than the current state-of-the-art method, while Precision was not significantly different. In conclusion, in this article we demonstrate that our approach, which is independent of axon tracing, can detect boutons to a high level of accuracy, and improves on the detection performance of existing approaches. The data and code (with an easy to use GUI) used in this article are available from open source repositories.

## Introduction

Imaging in neuroscience is widely used to study dynamic processes such as structural and functional neuronal plasticity. Three-dimensional, high-resolution images of neurons from many different modalities, including confocal, two-photon, and light-field microscopy, and many different species produce different types of data at a fast pace. For these datasets to be useful and for these studies to be reproducible there is the urgent need for robust analytical tools.

Over the last few years, there has been a significant effort to automate image analysis in neuroscience. For example, the Big Neuron project [1] is a community effort to standardise the tracing of neuronal structures [2–6]. However, while there have been some efforts at synapse detection and quantification, this has mainly focused on dendritic spines [7–11], the postsynaptic sites of synaptic connections. Several studies have shown an association between dendritic spine remodelling and motor learning [12], and a causal link has been recently established between spine enlargement and Long Term Potentiation (LTP), a process often associated with the formation of new memories [13, 14]. Axons are thinner and orders of magnitude longer than dendrites and as a result axonal boutons, i.e. the presynaptic sites, have received comparatively less attention [15, 16]. Improved analysis algorithms tailored for axonal boutons are urgently needed to deepen our understanding of the synaptic basis of behaviour and cognitive impairment [17, 18].

Manual methods like those provided in ImageJ [19–21], a software package that is often used in neuroscience, are the current standard to analyse axonal bouton data. The biggest limitations of manual analysis techniques are that results may differ between users, are time consuming, and can lead to bias. Even recent advances in semi-automated methods [22] such as EPBscore [16, 18] have limitations, EPBscore uniquely allows the analysis of boutons over time by first tracing the axon backbone, and then by using these traces to detect the locations of boutons. The tracing process is automated for relatively simple axons and good signal-to-noise conditions, but there are often more complex datasets that require a user’s input. There are other problems associated with relying on axon backbone tracing. For example, EPBscore typically fails to completely track axons with high curvature and apparent gaps due to low intensity, as well as those with several axons or branches (in the test dataset associated with this paper, when there are 2 or more axons or branches, 49% of them are partially detected, and only 10% of them are completely detected). Generally speaking, semi-automated tracing requires additional user input, which increases the time required for analysis. Finally, the EPBscore bouton detection algorithm uses the intensity profile of the traced axon. When the automatic tracing of an axon fails, boutons along those axons will be missed, wasting potentially meaningful data.

To overcome these limitations, we propose a fully automated bouton detection algorithm that is independent of the tracing of the axon. The proposed algorithm uses an interest point detector for the detection of candidate regions of interest (ROI); within these regions, visual features are extracted. These features are used to classify the regions as bouton or non-bouton using a Support Vector Machine (SVM) classifier. This approach improves on existing methods, allowing a faster, more accurate and reliable analysis of axonal boutons.

## Materials and methods

### Image acquisition and experimental details

#### Dataset

**Fig 1:**
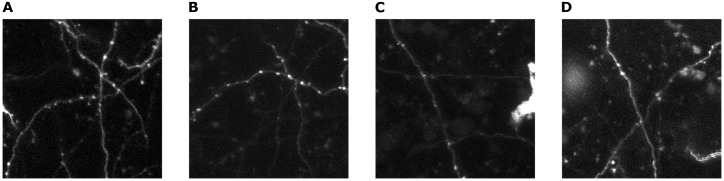
Examples from the 3D axon dataset. **A**, image with several crossing axons. **B**, image with 2 crossing axons with low intensity. **C**, image with high intensity noise (on the right). **D**, image with blob-like noise.

This Dataset consists of 100 images (TIFF files), split in 20 test and 80 training images, containing axons with their synapses (boutons) labelled. The labels are in form of ground-truth binary images of the same size, in which the corresponding synapses have been labelled as boxes. For examples of images from this dataset, see Fig 1. The data presented in this study is pre-existing data collected for other experimental studies, which are currently unpublished.

#### Animals

Images were collected from male mice (*n* = 6, 22 − 24 months old, and *n* = 8, 3 − 5 months old) from Thy-1-GFP-M, with cytosolic targeted GFP with C57BL/6 genetic background. Mice were housed in groups of two to four litter mates, in standard individually ventilated cages, and were maintained in a 12-h light-dark cycle with access to food and water ad libitum, with shelter and cardboard tunnels for environment enrichment. A long-term maintenance diet (R05-10; Scientific Animal Food and Engineering) was used to limit obesity. The animal work was approved by the local Animal Welfare and Ethical Review Body (AWERB). All experiments were conducted by trained researchers (Cher Bachar, and Vincenzo De Paola) holding a UK personal license, under a project license (70/7845), and in accordance with the Animals (Scientific Procedures) Act 1986 (United Kingdom) and associated guidelines.

#### Surgery

Cranial windows were surgically implanted overlying the barrel cortex (center coordinates were: Anterior Posterior −1.8 mm, and Lateral +2.8 mm from bregma) according to previously described methods [18, 23]. Briefly, mice were anesthetized with a ketamine-xylazine i.p. injection (0.083 mg/g ketamine, 0.0078 mg/g xylazine). The animals then were administered i.m. dexamethasone (0.02 mL at 4 mg/mL) to limit inflammation response and s.c. bupivacaine (1 mg/kg), a long-lasting local anesthetic. Once the skull was exposed, a few drops of the short-lasting local anesthetic lidocaine [1% (wt/vol) solution] were applied on its surface. The glass coverslip that seals the window was placed directly over the dura and the bone edges, with no agarose in between, and was sealed with dental cement. Mice were allowed to recover for 2-3 weeks before the start of the imaging protocol. Post-operative care was done by personal license holders, and trained staff at the animal house. Animals were monitored continuously for the first hour after surgery, daily until fully recovered (minimum 72 hr) and then at least three times a week after that to ensure no adverse effects are seen. Post operation analgesia was given (e.g. buprenorphine) within 72 hours post surgery, as advised by the Named Veterinary Surgeon (NVS).

A number of humane end points were observed, and mice were sacrificed by a schedule 1 method or non schedule 1 method, if 2 or more of the following clinical signs are present: piloerection, hunched posture, reduced activity, increased docility or aggression, weight loss up to 20% of body weight, dehydration persisting for 24 hours in spite of fluid replacement therapy, altered respiration, or self-mutilation.

#### In vivo imaging

Imaging was performed in a manner similar to Grillo et al [18, 23], with a few minor alterations, described below. A two-photon microscope equipped with a tunable Coherent Ti:Sapphire laser and Prarie software for image acquisition was used for all imaging experiments. Mice were anesthetized with Isoflurane, an inhalation anaesthetic, and secured to a fixed support under the microscope. To prevent dehydration in the eyes, Lacri-lube (Allergan) was applied. To regulate body temperature (37°C) an underlying heat pad was used and rehydration administered with isotonic saline solution (i.p.) as required during long imaging sessions. Depth of anesthesia was closely monitored by a video camera and regularly checking reflexes (toe pinch) and respiratory rates. An Olympus 4× with a 0.13 numerical aperture (NA) objective was used to identify characteristic blood vessel patterns, in order to relocate axons used in previous imaging sessions. An Olympus 40 × 0.80 NA water-immersion objective was used to acquire the images (512 × 512 pixels, 0.147 *μ*m per pixel for the *x*, *y* planes, and 1 *μ*m for the *z* plane. A Point Spread Function characterised by a Full Width at Half Maximum (FWHM) values of 0.45 × 0.45 × 2.5 *μ*m (*x*, *y*, *z*)). Either a water repellent pen or Vaseline (pure Petroleum Jelly) was applied around the cranial window to stabilize the meniscus for the 40x objective. A pulsed 910 nm laser beam was used never exceeding 70 mW on the back focal plane. Each imaging session typically lasted for 60–90 min, during which time up to 40 image stacks were collected.

### Algorithm details

#### Overview of bouton detection method

**Fig 2:**
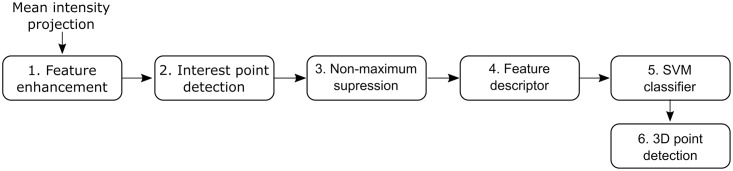
Flow chart of the bouton detection method. Our proposed algorithm has 5 main steps. (1) A negative Laplacian of Gaussian (LoG) mask is used in order to enhance blob-like objects (i.e. boutons) in the mean intensity projected image. (2) An interest point detector then detects the possible bouton locations. (3) Non-maximum suppression is used to move candidate boutons to their local maxima, and removes multiple detections of the same bouton in a close local area. (4) Feature vectors (with 12 elements each) are then generated at the location of the detected interest points. (5) A trained SVM classifies the points as boutons or non-boutons. (6) The last step uses the 2D coordinates to define a search volume for the 3rd coordinate.

To make use of the varicosity of boutons we decided on the following architecture. Our bouton detection algorithm consists of feature enhancement which strengthens the bouton-specific signals that are applied to the mean intensity projections of two-photon stacks. Following this, an interest point detector is used to identify candidate boutons, and a feature vector is constructed for each region of interest (ROI). With the local maxima of boutons increased by the blob feature enhancement module, the interest point detection module identifies candidate boutons, which significantly improves the scores (precision, recall, F1 score) and computation speed. In addition, a custom local maxima suppression algorithm is used in order to move candidate boutons to their local maxima. Candidate boutons are then classified as boutons and non-boutons using an SVM based on the extracted features. Lastly, we extend the 2D locations detected to 3D. In the process of choosing the most appropriate interest point detector and feature descriptor, we compared several well-known approaches and chose the best methods for our algorithm. A summary of the main steps of the algorithm is shown in Fig 2.

#### Feature enhancement module

**Fig 3:**
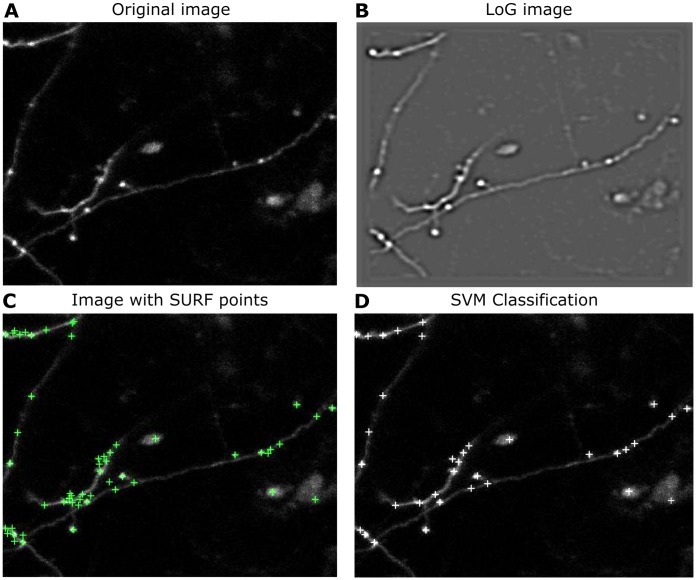
Example of the bouton detection method step by step. **A**, A mean intensity projection image from the 3D axon dataset. **B**, The same image convolved with LoG mask. **C**, In this example Interest points were detected using SURF (green “+” signs). **D**, Following SVM classification, the final proposed boutons are plotted on the mean projection image (white “+” signs).

The purpose of the Feature Enhancement module is to enhance structures corresponding to boutons (Fig 3). Because the varicosity of the boutons yields a blob-like image structure, we decided to use a negative Laplacian of Gaussian (LoG) convolution kernel with a set radius, as it has been shown to enhance such types of structure well [24]. The Gaussian kernel with a scale parameter, *σ*, is defined as

$$\begin{array}{c} G\left(x,y;\sigma \right)=\frac{1}{2\pi \sigma^{2}}\exp\{\frac{-(x^{2}+y^{2})}{2\sigma^{2}}\} \end{array}$$ (1)

where *x* and *y* denote spatial position in the imaging plane, and *σ* is the standard deviation of the Gaussian function *G*(*x*, *y*; *σ*). The LoG kernel may be thought of as a “dark blob” detector and is expressed as

$$\begin{array}{c} LoG\left(x,y;\sigma \right)=G_{xx}\left(x,y;\sigma \right)+G_{yy}\left(x,y;\sigma \right)=\frac{x^{2}+y^{2}-2\sigma^{2}}{\sqrt{2\pi \sigma^{5}}}\exp\{\frac{-(x^{2}+y^{2})}{2\sigma^{2}}\} \end{array}$$ (2)

at a certain scale space representation *LoG*(*x*, *y*; *σ*), where *G*_*xx*,*yy*_ denotes the partial second order derivatives in *x* and *y*, respectively. By converting to polar coordinates and considering only radial distance, *ρ*, (because *LoG*(*x*, *y*; *ρ*) is circularly symmetric), the value of *σ* that gives the maximum response for an image, *I*(*x*, *y*), containing light blobs with a radius, *r*, is such that:

$$\begin{array}{c} \frac{d}{d\sigma }[-\int_{0}^{r}LoG\left(\rho ;\sigma \right)d\rho ]=0 \end{array}$$ (3)

This leads to:

$$r^{2}-2\sigma^{2}=0$$

and so

$$\sigma =\frac{r}{\sqrt{2}}$$

Convolution of the image with the LoG kernel increases sensitivity of the bouton detection by accentuating local extrema corresponding to blob-like structures in the image. In our case, we use the prior value for *σ* = 4, which reflects the average diameter of a bouton (average of 20 boutons from this dataset, *σ* = 4.56 pixels; 512 × 512 resolution; 0.147 *μ*m per pixel). Convolving the image with −*LoG*(*x*, *y*; *σ*) suppresses the intensity of image structures significantly smaller than *σ*. By moving to a coarser scale image, bouton detection specificity is increased with the disappearance of small structures that could become false positive instances of boutons in the next step of processing (Fig 2). This is particularly important, due to the scale-invariance of the succeeding interest point module for detecting candidate boutons. Moreover, the smoothing effect of the LoG kernel increases the stability of the subsequent interest point detection by reducing shot noise, and increasing the image response of blob-like structures. One disadvantage of this is that it may lead to duplicate detections of the same bouton. To address this, a custom non-maximum suppression step is used.

#### Interest point detection module

The purpose of using an interest point detector is to find candidate locations for the boutons within the image. This allows the remainder of the bouton detection process to focus on using computationally expensive routines to separate the candidate boutons from non-bouton structures, far more efficient than pixel-wise application of a feature descriptor (see Feature Descriptor module). We selected three widely used interest point detectors (Harris, SURF keypoint detector and SIFT keypoint detector), focusing on those that are more likely to be appropriate for detecting the blob-like nature of boutons. We explain the reasons for these choices below, evaluating their performance in a later section.

The Harris corner detector relies on the principle that at a corner, the image intensity within a local window will change considerably when the window is shifted in different directions [25]. Because a bouton is a Gaussian-like object [26] that changes its intensity along all radial directions, it displays some attributes that might be considered to be corner-like. The Harris detector makes use of local partial derivatives in intensity over space, computing a 2 × 2 Harris matrix [25]. The eigenvalues of the matrix yield a corner response at all locations in the image, which is then thresholded to produce candidate bouton locations. We used the *Matlab* implementation of the Harris detector in this work.

The SIFT interest point detector forms one part of the SIFT keypoint detector [27], which is in extremely wide usage in object recognition, image stitching and video tracking [28, 29]. The interest point locations of SIFT are identified by finding local maxima in a scale-space representation of the original image; this representation is obtained by blurring the image with Gaussian masks at different spatial scales. We used the VLFEAT implementation of SIFT [30].

Finally, the SURF keypoint detector [31] uses box filter approximations of second order spatial Gaussian functions in order to construct a local Hessian matrix; the determinant of this matrix can be used to find locations of intensity minima, maxima and saddle points. The approach has been suggested [31] to be good at dealing with slightly elongated structures, making it suitable for detecting the possible locations of boutons, particularly since they sit on or very near to axons. We used the *Matlab* implementation of SURF.

#### Non-maximum suppression module

In this step, we propose a way to eliminate candidate bouton locations that are present in a relatively close local area by using non-maximum suppression [32]. This is due to the statistics of the image data, and tendency of SURF interest point detector to fire several times around circular structures, which are common in microscopy data. By removing multiple detections of the same bouton automatically, the user will not need to manually remove the over-detections.

In this paper, non-maximum suppression is done differently to that of the Canny edge detector in [32], as it is applied to candidate bouton centres rather than candidate edge locations. To place candidate boutons at their local maxima, we iterate over all candidate bouton locations and relocate them to the pixel with the highest intensity in their local area (= *W*_1_ pixels). In order to save time in manual user intervention, we then remove all boutons in the local area (= *W*_2_ pixels), apart from one candidate bouton in the location with highest pixel intensity. We found that the best values for *W*_1_ and *W*_2_ were 20 and 10 respectively.

#### Feature descriptor module

The purpose of using a feature descriptor is to capture the appearance of image regions around candidate bouton locations; these descriptors are then used as observation vectors to classify the candidate interest points as boutons or non-boutons. In this section, we briefly describe 3 feature descriptors that were evaluated for this task; one of these is custom designed for the bouton detection task.

#### Standard region descriptors

The more widely used feature descriptors (including SIFT [27] and HOG [33, 34]) for local image appearance consist of a standard series of steps, based around estimating the local intensity gradient field around a reference location (such as an interest point): they differ mainly in the local operators used to estimate the intensity gradient field, the way that the gradient field is encoded in to the descriptor, and the way that the distributions of gradient orientation are normalised. We used the most widely employed standard implementations of HOG (*Matlab*) and SIFT-based descriptors (VLFEAT [30]). HOG yields a 144 element descriptor which describes the gradient field in a number of square pixel regions arranged around the interest point. SIFT yields a 128 element rotation invariant description of a center-weighted local image gradient field, again computed around the interest point. We reduced the length of both descriptors to 12 elements by using minimum Redundancy, Maximum Relevance [35, 36]. This process selects the most informative and least redundant subset of features, speeding processing, and improving stability. Because reducing dimensionality in this way changes the normalisation of the remaining elements of the vector, we re-normalise the reduced vectors using the best of *L*_1_, *L*_2_ and *L*_∞_ normalisation on the remaining elements within the validation data set.

#### A custom descriptor

SIFT descriptors and the HOG descriptor are designed for general use in visual recognition. We hypothesized that a custom-designed descriptor, providing a joint encoding of spatial and directional structure, could improve the ability of a classifier to distinguish boutons from non-boutons. Accordingly, we built a Gabor-based feature descriptor that was designed to perform well for the bouton detection problem.

**Fig 4:**
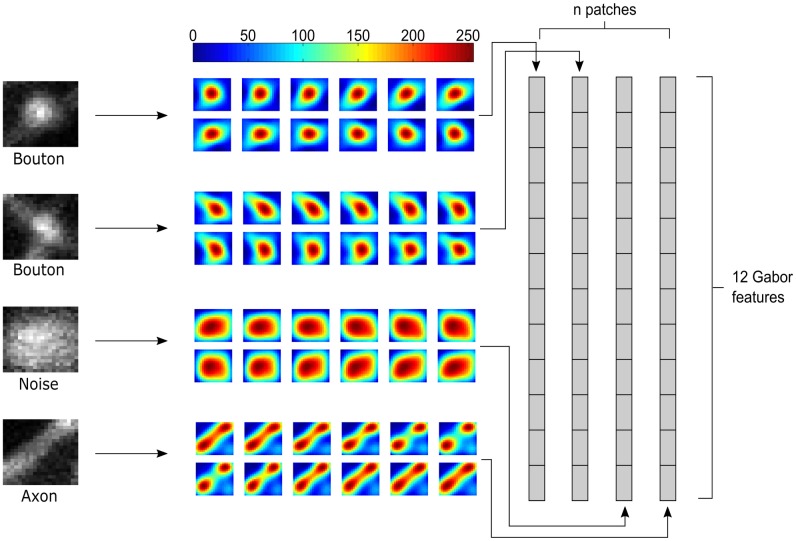
Example of bouton features generated using Gabor filters. On the left, there are examples of 4 different types of patches extracted from the interest points (in this example, by the SURF detector). The first 2 are examples of boutons, the 3rd is an example of noise, and the 4th an example of an axon segment. The image patches are then convolved with 12 different Gabor filters and their inner product is computed to create a 12-dimensional feature vector. The colorbar shows the pixel intensities.

Gabor filters give large responses at intensity edges and within regions of an image where the texture matches that of the filter. They have successfully been used for edge detection [37] and texture segmentation [38, 39]. However, the parameters of spatial Gabor filters can also be tuned so that they yield strong responses to blob-like structures. The patterns of intensity around boutons also tend to contain some directional structures (e.g. the axons they lie on). Thus, without performing explicit axon detection, a descriptor that takes the visual of structure surrounding boutons into account can be constructed from patterns of directional Gabor filters. The two-dimensional Gabor functions were selected using the standard definition for symmetric 2D Gabor functions:

$$\begin{array}{c} g_{even}\left(x,y\right)=\frac{1}{2\pi \sigma_{x}\sigma_{y}}\exp[-\frac{1}{2}[\frac{{\left(y-y_{0}\right)}^{2}}{\sigma_{y}^{2}}+\frac{{\left(x-x_{0}\right)}^{2}}{\sigma_{x}^{2}}]]\cos\left(2\pi w_{x_{0}}x+2\pi w_{y_{0}}y\right) \end{array}$$ (4)

where *σ*_*x*_ and *σ*_*y*_ are the standard deviations of the elliptical Gaussian for *x* and *y*, respectively. The centre of the receptive field in the spatial domain is (*x*_0_, *y*_0_) and ($w_{x_{0}},w_{y_{0}}$) is the optimal spatial frequency of the filter in the frequency domain. The Gabor filters module uses several Gabor filters with varying spatial frequency and standard deviations to construct a robust bouton discriminatory feature vector **x** = [*x*_1_, …, *x*_*V*_]. Element *d* of the feature vector **x** is defined as:

$$\begin{array}{c} x_{d}=\sum_{i=0}^{I-1}\sum_{j=0}^{J-1}f\left(i,j\right)\cdot g_{d}\left(i,j\right) \end{array}$$ (5)

where *f* is an image patch of size *I* × *J* pixels meant to capture the entirety of individual boutons and has its centre in a single interest point. The inner products between an image patch and the Gabor filters at angles *θ* = *nπ*/6, {*n* = 1, 2, 3, …, 12} make up the feature vector **x** with *V* = 12 features (Fig 4). The image patch size is determined by the standard deviation used in the interest point detection module such that

$$\begin{array}{c} \frac{I-1}{2}=3\sigma_{x} \end{array}$$ (6)

$$\begin{array}{c} \frac{J-1}{2}=3\sigma_{y} \end{array}$$ (7)

because *σ*_*x*_ and *σ*_*y*_ are approximately equal to 4, and *f* consists of 25 × 25 pixels.

#### SVM classification module

The classification module is trained to identify the true positives among candidate bouton regions (obtained from the interest points) using the extracted features (Fig 3G). For this detection task, we use a SVM, which is a sparse kernel machine that maximises the margin between points of different classes in a high-dimensional feature space. SVMs have been found to be easy to train, are widely implemented and are relatively fast in classifying. Our SVM uses a polynomial discriminant function to maximise the margin between the two classes of candidate boutons. The regularisation coefficient, *C* > 0, penalises the misclassification of non-boutons as boutons and vice versa. To train and validate the SVM to correctly classify interest points, 80 images were used; 900 patches were randomly selected for training and validation. The collected training image patches of negative and positive instances of boutons were resized to 25 × 25 pixels to match the size of the average bouton. All the training images contain a part of an axon.

#### 3D point detection module

The 3D point detection module aims to transfer the final 2D points on the mean intensity projection image detected by the algorithm, and extend them to 3D (i.e. detect which slice they lie on).

For each bouton detected in 2D, a 3D point is found by:

$$\begin{array}{c} z=\underset{k}{\arg\max}\sum_{i=1}^{N}\sum_{j=1}^{N}f\left(x_{i},y_{j},z_{k}\right) \end{array}$$ (8)

In which *f*(*x*_*i*_, *y*_*i*_, *z*_*k*_) is a voxel region in 3D space, *k* is the *z* coordinate, *x*_*i*_, *y*_*i*_ are the (*x*, *y*) coordinates, *N* is the number of pixels in a patch. For each bouton detected, the algorithm iterates over all slices in the stack, and sums the pixel intensities within a 25 × 25 patch centred on the point. The slice with highest overall value is chosen as the location *z*.

#### Method validation

We compared the performance of the algorithm to manual labelling of boutons, done by 3 expert neurobiologists, and another automated tool, *EPBscore*, a *Matlab*-based software tool available for detection and analysis of axonal boutons. We used a total of 100 images in the training, validation (80 images), and testing (20 images). The images chosen for testing had a few of axons per image, and considerable noise (Fig 1). Each image had a size of 512 × 512 × *Z* voxels, ∈[15, 50]. We used 900 image patch examples of boutons and non-boutons (450 each) for training (720 patches) and validation (180 patches). A total of 345 (average of 17 per image) boutons were present in the test images.

**Fig 5:**
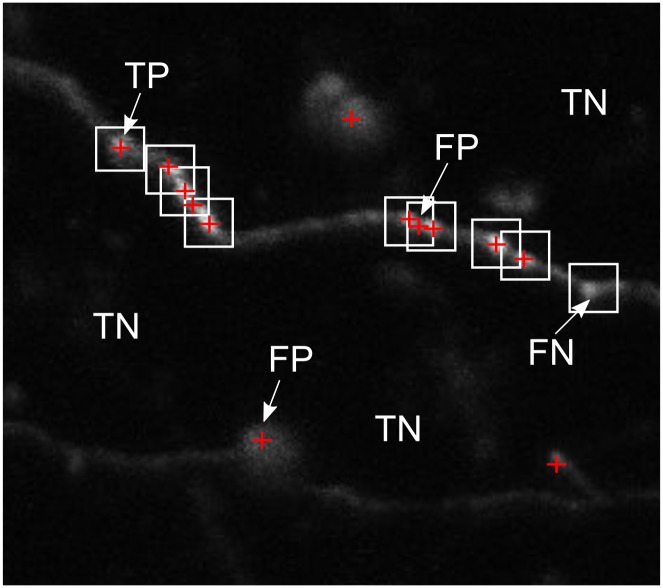
Examples of how True Positives (TP), False Positives (FP), False Negative (FN) and True Negatives (TN) were classified. For the calculation of these scores, we manually labelled boxes around the correct boutons. A point was classified as a TP only when its *x* and *y* coordinates lie within one of the boxes, and only 1 TP was counted per box (i.e. if there are 2 points within the box, 1 would be counted as a TP, and one would be counted as FP). FNs were classified for the number of detected boxes that did not have any points. The TNs were all the other points in the image (not including the 25 × 25 boxes around all other TPs, FPs, and FNs).

To compute the metrics (Precision, Recall, F1 score and AUC), we calculated the amount of True Positives (TP), True Negatives (TN), False Positives (FP) and False Negatives (FN) that were present in each image (Fig 5). Precision is computed using TPs and FPs ($Precision=\frac{TP}{TP+FP}$), Recall is computed using TPs and FNs ($Recall=\frac{TP}{TP+FN}$), and F1 score is described by both Precision and Recall ($F_{1}=2\times \frac{precision\times recall}{precision+recall}$).

## Results

### Comparison of feature descriptors and interest point detectors

**Fig 6:**
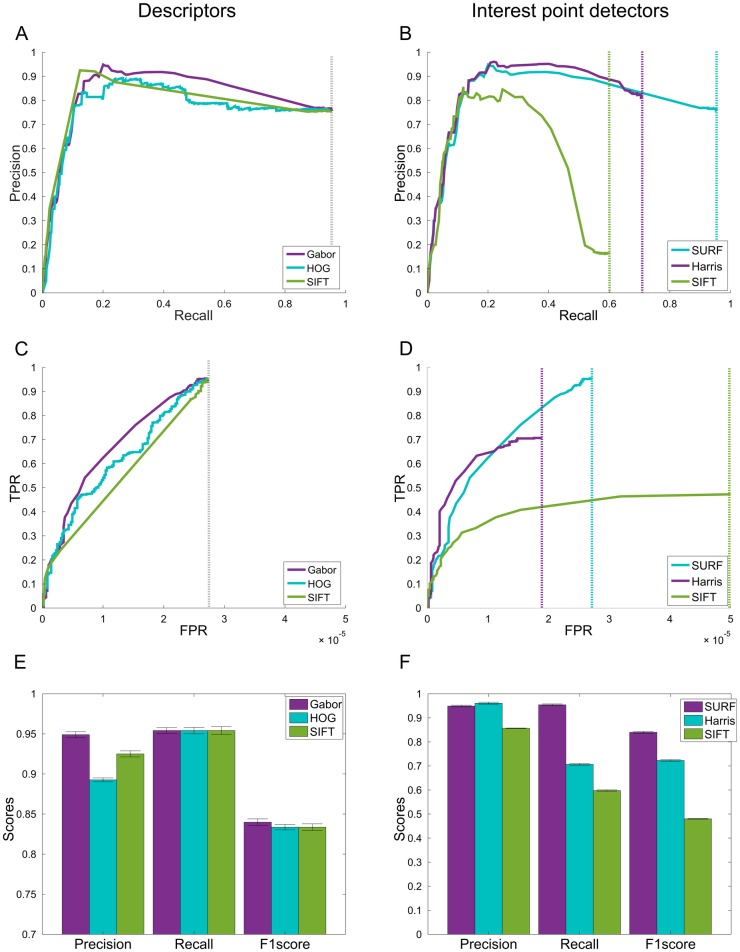
Graphs comparing the performance of the descriptors and interest point detectors at 10^3^ SVM class thresholds. We chose Gabor and SURF, as our descriptor and interest point detector, as they had better performance than the other methods (all with separately optimized hyperparameters). The precision-recall graphs seem to have an unusual curvature; however, this can be explained by the nature of the dataset. In this axon dataset, where the number of TPs (i.e. boutons) is relatively small compared to the size of the image, it is to be expected that there will always be some FP detections when TP points are also detected. As such, there will never be a case in which precision = 1, as there will always be some FPs detected as well (i.e. the SVM can not have a FPR of 0). **A**, Precision-Recall curve comparing feature descriptors (AUC: Gabor = 0.779, HOG = 0.728, SIFT = 0.75). Gabor based descriptors reached the highest Precision, and has the best overall performance, demonstrated by the AUC. **B**, Precision-Recall curve comparing interest point detectors (AUC: SURF = 0.779, Harris = 0.598, SIFT = 0.357). SURF reaches the best TPR in comparison to the other methods. **C**, ROC curve comparing feature descriptors (AUC: Gabor = 1.8 × 10^−5^, HOG = 1.65 × 10^−5^, SIFT = 1.49 × 10^−5^). Gabor has the best overall performance, demonstrated by the AUC. **D**, ROC curve comparing interest point detectors (AUC: SURF = 1.8 × 10^−5^, Harris = 1.08 × 10^−5^, SIFT = 4.69 × 10^−6^). SURF reaches the best Recall in comparison to the other methods. **E-F**, Error bar graphs comparing metrics between the descriptors and interest point detectors, respectively. Gabor and SIFT have the best overall performance across the metrics compared. The dotted lines are where the graphs saturate. TPR, True Positive Rate; FPR, False Positive Rate; FP, False Positive; TP, True Positive; Error bars, SEM; AUC, Area Under Curve.

We compared several well-known feature descriptors (Fig 6, left), including HOG, and SIFT, and Gabor kernels. We trained the SVM using the same datasets on HOG, SIFT, or Gabor features. For a fair comparison with the Gabor features, we extracted the 12 most significant features for each by using minimum redundancy feature selection [35, 36], and normalized the features using the best normalization method for each descriptor (*L*_1_, *L*_2_, *L*_∞_). We also optimized the SVM hyperparameters (e.g. Kernel function, Polynomial Orders (*d*), Cost (*C*), and *γ*) and used the best result for each descriptor. Comparison between the descriptors is shown in Fig 6. We compared various performance measures including Precision, Recall, F1 score, and Area Under Curve (AUC).

The proposed Gabor-based descriptor had the best performance compared to both HOG and SIFT (Fig 6, left). We also compared several interest point detectors (SURF, Harris and SIFT), as an improvement in the interest point detection can have a big influence on the final performance of the algorithm. SURF had significantly better Recall and F1 scores (Fig 6, right). We therefore chose SURF as our interest point detector, and Gabor as our feature descriptor. In a later section, we compare our method against the existing semi-automated alternative for bouton detection (within EPBscore [16]).

### Optimization of hyperparameters

To optimize the performance of the algorithm, we compared the Precision-Recall and Receiver operating characteristic (ROC) curves for different descriptors and interest point detectors (Fig 6) on a validation set. After scaling the SVM scores to range [−1, 1], we found that the threshold—defining a point on the ROC—that gave the best results was at −0.0399, giving a mean F1 score of 0.840 (Precision = 0.765; Recall = 0.952). We optimized our algorithms after choosing the optimal normalization paradigm on the features, and on different SVM hyperparameters. These include kernel functions (Polynomial and Gaussian), polynomial orders, Cost (*C*), and *γ*.

Our best performing SVM model has a polynomial kernel (order = 3) and a regularisation term that penalises the misclassification of non-boutons and boutons.

### Comparison to EPBscore

**Fig 7:**
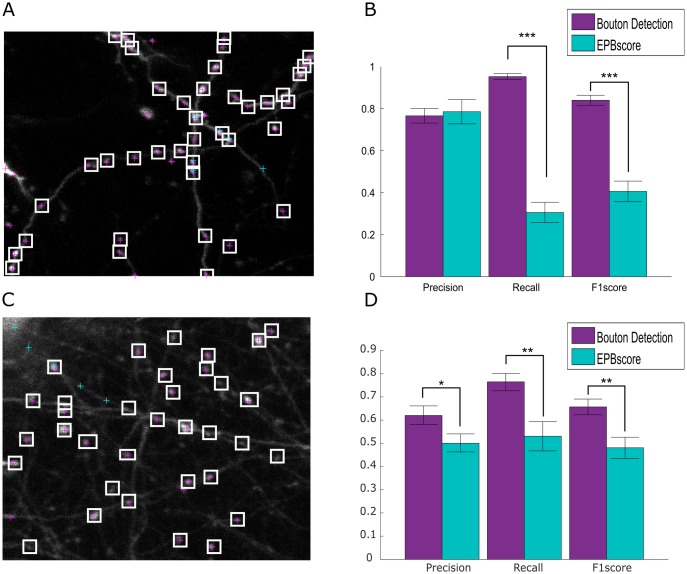
Comparative scores of our bouton detection algorithm versus EPBscore results. Results of detection in EBPscore versus our algorithm in the test dataset (**A-B**), and in a published dataset (**C-D**) [23]. **A**, Example of bouton detection in the test dataset. The white boxes indicate the true positive boutons, and the purple crosses are the boutons detected by the algorithm, and green/ teal crosses are boutons detection by EPBscore. **B**, The proposed bouton detection method has significantly better Recall (*p* < 10^−5^, KS test) and F1 (*p* < 10^−5^, unpaired two-tailed *t*-test) scores than EBPscore. **C**, Example of bouton detection in the published dataset. **D**, In the published dataset, the proposed bouton detection method has significantly better Precision (*p* = 0.04), Recall (*p* = 0.002, unpaired two-tailed *t*-test) and F1 (*p* = 0.004, unpaired two-tailed *t*-test) scores than EBPscore. Error Bars, SEM; Kolmogorov-Smirnov, KS.

In this section, we compare our algorithm (using SURF and Gabor features, as our interest point detector and feature descriptor, respectively) to EPBscore. When analysing data from neurons, it is important to include as many synapses (i.e. boutons) as possible in the analysis. Hence, we used the measure of Recall (the number of true boutons that were correctly detected). We found that our algorithm detected boutons (TPs) extremely well (Fig 7, Table 1), with a 0.952±0.15 Recall compared to EPBscore that had a recall of 0.306±0.21 (*p* < 10^−5^, Kolmogorov-Smirnov (KS) test). The Precision, which is an indication of the number of false bouton detections, was also measured. There was no significant difference (*p* = 0.135, KS test) in the precision of our algorithm and EBPscore. We then computed the F1 score, which takes into account both Precision and Recall. Our detector is significantly better (*p* < 10^−5^, unpaired two-tailed *t*-test); with 0.433 higher average F1 score than EPBscore, demonstrating that our algorithm performs much better than the current state of the art method used to analyse synaptic boutons.

**Table 1:** Comparative scores of our bouton detection algorithm versus EPBscore results.

Image	Number of boutons	Bouton detection algorithm			EPBscore		
		Precision	Recall	F1-score	Precision	Recall	F1-score
1	10	0.71	1.00	0.83	1.00	0.20	0.33
2	8	1.00	1.00	1.00	1.00	0.25	0.40
3	27	0.73	1.00	0.84	0.70	0.26	0.38
4	40	0.85	0.98	0.91	0.83	0.13	0.22
5	26	0.74	1.00	0.85	0.80	0.77	0.78
6	16	0.56	0.94	0.70	1.00	0.75	0.86
7	11	0.91	0.91	0.91	0.70	0.64	0.67
8	7	1.00	1.00	1.00	0.00	0.00	0.00
9	4	0.67	1.00	0.80	0.50	0.25	0.33
10	10	0.91	1.00	0.95	1.00	0.20	0.33
11	11	0.92	1.00	0.96	0.57	0.36	0.44
12	9	0.78	0.78	0.78	1.00	0.22	0.36
13	25	0.78	0.84	0.81	0.71	0.20	0.31
14	49	0.66	0.88	0.75	1.00	0.08	0.15
15	31	0.88	0.90	0.89	0.73	0.35	0.48
16	12	0.71	1.00	0.83	1.00	0.50	0.67
17	17	0.45	1.00	0.62	1.00	0.47	0.64
18	9	0.53	0.89	0.67	0.67	0.22	0.33
19	8	0.89	1.00	0.94	0.50	0.13	0.20
20	15	0.64	0.93	0.76	1.00	0.13	0.24
Average	17.25	**0.76**	**0.95**	**0.84**	**0.79**	**0.31**	**0.41**
STD		0.15	0.06	0.10	0.25	0.21	0.21

On average, Recall and F1 score were better by 0.65 and 0.43, respectively (*p* < 10^−5^). Precision is not significantly different (*p* = 0.135) between the methods.

### Application to a published dataset

We applied our algorithm to a published dataset from De Paola et al [23], in which they did a morphological analysis of axons, and quantified synaptic dynamics. The data was collected using a two-photon microscope, in the mouse barrel cortex. The genetic background was Thy-1 transgenic mice in a c57/bl6 background, expressing cytoplasmic GFP (more details of experimental method in [23]). We analysed 22 images, containing 348 boutons.

The algorithm performed well, even without being tuned to this dataset. Example of performance is shown in Fig 7C. Precision (*p* = 0.04, unpaired two-tailed *t*-test), Recall (*p* = 0.002, unpaired two-tailed *t*-test) and F1 (*p* = 0.004, unpaired two-tailed *t*-test) scores were significantly better than EBPscore (Fig 7D).

## Discussion

We used an algorithm based on interest point detection, feature extraction, and classification using an SVM. In our chosen method, first a negative LoG mask was convolved with the mean projection image, which produced an image with enhanced blob-like objects (i.e. boutons). Then a SURF point detector was applied to the pre-processed LoG image, which extracts interest points for the classifier. These image patches [25 × 25 pixels] were convolved with 12 Gabor filters to generate a feature vector for each interest point. The feature vectors were then processed by the SVM classifier which gave the final detected 2D bouton locations, which can be extended to 3D locations.

We found that our algorithm is significantly better (*p* < 10^−5^) than the current state of the art method (EPBscore) in the same dataset. It detects nearly all boutons that were present in the images, with a mean Recall of 0.95 ± 0.06. The limitation of the algorithm is that, in our tests, it sacrifices a small amount of precision (i.e. more FPs) for a better recall. Most of the FPs were either due to noise, spines or intersection points in the axons. However, an algorithm that is independent of the axon or neuron tracing is advantageous, as it is faster, and reliably produces likely bouton candidates, unlike methods based on neuronal tracing which can be slow, manual, and produce variable results that are dependent on the neuron trace. To remove the extra FPs, we include a simple Graphical User Interface (GUI) that allows a user to easily remove these over-detected points, or to add undetected boutons. The GUI also records these FPs, so that they can be used in future training.

The algorithm presented in this paper is likely to work best for images acquired using a similar protocol to the dataset provided here. Indeed, we demonstrated that the algorithms worked well in another published dataset [23], which had similar conditions (Fig 7C and 7D). It is also likely to work well for other image datasets with similar blob-like objects, because of the data this algorithm was trained on, as well as the steps taken in the algorithm to detect initial points. However, is it possible that other imaging protocols with considerably different conditions might lead to different contrasts, resolutions, and noise levels which might require changes to some of the modules, or require additional training for optimal results. In addition, the use of interest point detectors also provides scale estimates that could be used to support the detection of boutons at different magnification factors, either through training the classifier with the scale estimate, or using the scale estimate to tune the Gabor parameters. We suggest this for future work.

The strategy for detecting boutons on the mean intensity projection of a stack, and then tracing back through slices to determine the *z* coordinate, works well for the acquisition protocol used in this and similar studies, and requires low computational effort. However, there is the small possibility that axons which cross in the third dimension (*z*), and have boutons in a nearby *x*, *y* location, can lead to incorrect bouton detections. The solution to this problem lies in creating a fully three-dimensional bouton detector which is able to operate on the 3D data by taking into account the slice spacing and slice thickness of the confocal stack. Future work will involve the use of intensive training using surrogate data in order to learn a 3D bouton detector. This might employ a deep learning architecture, currently a very popular class of methods in computer vision, which has also been applied to biomedical image analysis [40–44]. Deep learning methods are often trained end-to-end, i.e. the features that are learned during training are not hand crafted, and are optimal for the particular task in hand. This kind of approach might yield improved performance over methods with hand-crafted features. It is, however, achieved at the expense of having sufficiently large numbers of datasets with labelled boutons. The benefit of the current approach is that the parameters are sufficiently small in number to enable hand optimization.

We will also extend the analysis to time-series images in order to analyse the changes in synapses over time, analysis often required in the study of structural plasticity of neurons.

## Conclusion

In this paper we proposed an algorithm for the detection of axonal boutons in 3D two-photon microscopy images. We found that using SURF keypoints and Gabor features gives the best results after comparing several well-known keypoint detectors and feature descriptors, and that the algorithm provides significant improvements over the currently available methods. Most importantly, our method makes advances in automated bouton detection without tracing the axons first, which can be an inaccurate and a computationally expensive step. We showed that despite removing this step, a high Recall of 95% is achieved, therefore detecting more true boutons than the existing method [16]. Increasing the TPR is an important factor for the analysis of axonal boutons in neuroscience research, as it can significantly increase the number of bouton samples that can be analysed. Since this type of data is often limited and hard to acquire, it is important to detect all boutons present in the image to get more data for the statistical analysis. This usually requires a significant and time-consuming amount of user-intervention, but by using the approach presented in this paper, it may be more easily achieved, as the vast majority of boutons are found by the algorithm.

## References

[1] Peng H, Hawrylycz M, Roskams J, Hill S, Spruston N, Meijering E, et al BigNeuron: Large-Scale 3D Neuron Reconstruction from Optical Microscopy Images. Neuron. 2015;87(2):252–256. 10.1016/j.neuron.2015.06.036 26182412 PMC4725298

[2] Chen H, Xiao H, Liu T, Peng H. SmartTracing: self-learning-based Neuron reconstruction. Brain Informatics. 2015;2(3):135–144. 10.1007/s40708-015-0018-y 27747506 PMC4883140

[3] Xiao H, Peng H. APP2: Automatic tracing of 3D neuron morphology based on hierarchical pruning of a gray-weighted image distance-tree. Bioinformatics. 2013;29(11):1448–1454. 10.1093/bioinformatics/btt170 23603332 PMC3661058

[4] Peng H, Long F, Myers G. Automatic 3D neuron tracing using all-path pruning. Bioinformatics. 2011;27(13):i239–i247. 10.1093/bioinformatics/btr237 21685076 PMC3117353

[5] Zhou Z, Liu X, Long B, Peng H. TReMAP: Automatic 3D Neuron Reconstruction Based on Tracing, Reverse Mapping and Assembling of 2D Projections. Neuroinformatics. 2016;14(1):41–50. 10.1007/s12021-015-9278-1 26306866

[6] Yang J, Gonzalez-Bellido PT, Peng H. A distance-field based automatic neuron tracing method. BMC Bioinformatics. 2013;14(19). 10.1186/1471-2105-14-93 PMC3637550 23497429

[7] Koh IYY, Lindquist WB, Zito K, Nimchinsky Ea, Svoboda K. An image analysis algorithm for dendritic spines. Neural Computation. 2002;14(6):1283–1310. 10.1162/089976602753712945 12020447

[8] Jammalamadaka A, Banerjee S, Kosik KS, Manjunath BS. Statistical Analysis of Dendritic Spine Distributions in Rat Hippocampal Cultures. BMC Bioinformatics. 2013;14(1):287 10.1186/1471-2105-14-287 24088199 PMC3871014

[9] Yuan X, Trachtenberg JT, Potter SM, Roysam B. MDL constrained 3-d grayscale skeletonization algorithm for automated extraction of dendrites and spines from fluorescence confocal images. Neuroinformatics. 2009;7(4):213–232. 10.1007/s12021-009-9057-y 20012509 PMC2844542

[10] Janoos F, Mosaliganti K, Xu X, Machiraju R, Huang K, Wong STC. Robust 3D reconstruction and identification of dendritic spines from optical microscopy imaging. Medical Image Analysis. 2009;13(1):167–179. 10.1016/j.media.2008.06.019 18819835 PMC2663851

[11] Rodriguez A, Ehlenberger DB, Dickstein DL, Hof PR, Wearne SL. Automated three-dimensional detection and shape classification of dendritic spines from fluorescence microscopy images. PLoS ONE. 2008;3(4). 10.1371/journal.pone.0001997 PMC2292261 18431482

[12] Fu M, Yu X, Lu J, Zuo Y. Repetitive motor learning induces coordinated formation of clustered dendritic spines in vivo. Nature. 2012;483(7387):92–95. 10.1038/nature10844 22343892 PMC3292711

[13] De Roo M, Klauser P, Muller D. LTP promotes a selective long-term stabilization and clustering of dendritic spines. PLoS Biology. 2008;6(9):1850–1860. 10.1371/journal.pbio.0060219 PMC2531136 18788894

[14] Kasai H, Fukuda M, Watanabe S, Hayashi-Takagi A, Noguchi J. Structural dynamics of dendritic spines in memory and cognition. Trends in Neurosciences. 2010;33(3):121–9. 10.1016/j.tins.2010.01.001 20138375

[15] Prodanov D, Heeroma J, Marani E. Automatic morphometry of synaptic boutons of cultured cells using granulometric analysis of digital images. Journal of Neuroscience Methods. 2006;151(2):168–77. 10.1016/j.jneumeth.2005.07.011 16157388

[16] Song S, Grillo FW, Xi J, Ferretti V, Gao G, De Paola V. EPBscore: a Novel Method for Computer-Assisted Analysis of Axonal Structure and Dynamics. Neuroinformatics. 2015; p. 9274. 10.1007/s12021-015-9274-5 26163988

[17] Canty AJ, Teles-Grilo Ruivo LM, Nesarajah C, Song S, Jackson JS, Little GE, et al Synaptic elimination and protection after minimal injury depend on cell type and their prelesion structural dynamics in the adult cerebral cortex. The Journal of Neuroscience. 2013;33(25):10374–83. 10.1523/JNEUROSCI.0254-13.2013 23785150 PMC6618592

[18] Grillo FW, Song S, Ruivo LMTg, Huang L, Gao G, Knott GW, et al Increased axonal bouton dynamics in the aging mouse cortex. PNAS. 2013;110(16):E1514–E1523. 10.1073/pnas.1218731110 23542382 PMC3631669

[19] Mostany R, Anstey JE, Crump KL, Maco B, Knott G, Portera-Cailliau C. Altered synaptic dynamics during normal brain aging. The Journal of Neuroscience. 2013;33(9):4094–104. 10.1523/JNEUROSCI.4825-12.2013 23447617 PMC6619332

[20] Becker N, Wierenga CJ, Fonseca R, Bonhoeffer T, Nägerl UV. LTD induction causes morphological changes of presynaptic boutons and reduces their contacts with spines. Neuron. 2008;60(4):590–7. 10.1016/j.neuron.2008.09.018 19038217

[21] Majewska AK, Newton JR, Sur M. Remodeling of synaptic structure in sensory cortical areas in vivo. The Journal of Neuroscience. 2006;26(11):3021–9. 10.1523/JNEUROSCI.4454-05.2006 16540580 PMC6673961

[22] Marik Sa, Yamahachi H, McManus JNJ, Szabo G, Gilbert CD. Axonal dynamics of excitatory and inhibitory neurons in somatosensory cortex. PLoS Biology. 2010;8(6):e1000395 10.1371/journal.pbio.1000395 20563307 PMC2885981

[23] De Paola V, Holtmaat A, Knott G, Song S, Wilbrecht L, Caroni P, et al Cell type-specific structural plasticity of axonal branches and boutons in the adult neocortex. Neuron. 2006;49(6):861–75. 10.1016/j.neuron.2006.02.017 16543134

[24] Kong H, Akakin HC, Sarma SE. A generalized Laplacian of Gaussian filter for blob detection and its applications. IEEE Transactions on Cybernetics. 2013;43(6):1719–1733. 10.1109/TSMCB.2012.2228639 23757570

[25] Harris C, Stephens M. A Combined Corner and Edge Detector. Procedings of the Alvey Vision Conference. 1988;15:50.

[26] Kitti T, Jaruwan T, Chaiyapon T. An Object Recognition and Identification System Using the Harris Corner Detection Method. International Journal of Machine Learning and Computing. 2012;2(4):462–465. 10.7763/IJMLC.2012.V2.168

[27] Lowe DG. Distinctive image features from scale invariant keypoints. International Journal of Computer Vision. 2004;60(2):91–110. 10.1023/B:VISI.0000029664.99615.94

[28] Mikolajczyk, Krystian; Schmid C. Indexing based on scale invariant interest points. Eighth IEEE International Conference on Computer Vision. 2001;1:525–531.

[29] Zhou H, Yuan Y, Shi C. Object tracking using SIFT features and mean shift. Computer Vision and Image Understanding. 2009;113(3):345–352. 10.1016/j.cviu.2008.08.006

[30] Vedaldi A, Fulkerson B. VLFeat: An Open and Portable Library of Computer Vision Algorithms; 2008. http://www.vlfeat.org/

[31] Bay H, Tuytelaars T, Van Gool L. SURF: Speeded up robust features. European Conference on Computer Vision. 2006; p. 404–417.

[32] Canny J. A Computational Approach to Edge Detection; 1986. 21869365

[33] Dalal N, Triggs B. Histograms of oriented gradients for human detection. IEEE Computer Society Conference on Computer Vision and Pattern Recognition. 2005;I:886–893.

[34] Zhang Y, Brady M, Smith S. Segmentation of brain MR images through a hidden Markov random field model and the expectation-maximization algorithm. IEEE Transactions on Medical Imaging. 2001;20(1):45–57. 10.1109/42.906424 11293691

[35] Peng HC, Long FH, Ding C. Feature selection based on mutual information: Criteria of max-dependency, max-relevance, and min-redundancy. IEEE Transactions on Pattern Analysis and Machine Intelligence. 2005;27(8):1226–1238. 10.1109/TPAMI.2005.159 16119262

[36] Ding C, Peng H. Minimum redundancy feature selection from microarray gene expression data. Journal of Bioinformatics and Computational Biology. 2005;3(2):185–205. 10.1142/S0219720005001004 15852500

[37] Mehrotra R, Namuduri KR, Ranganathan N. Gabor filter-based edge detection. Pattern Recognition. 1992;25(12):1479–1494. 10.1016/0031-3203(92)90121-X

[38] Jain aK, Farrokhnia F. Unsupervised texture segmentation using Gabor filters. Pattern Recognition. 1991;24(12):1167–1186. 10.1016/0031-3203(91)90143-S

[39] Weldon TP, Higgins WE, Dunn DF. Efficient Gabor filter design for texture segmentation. Pattern Recognition. 1996;29(12):2005–2015. 10.1016/S0031-3203(96)00047-7

[40] Xu Y, Li Y, Liu M, Wang Y, Fan Y, Lai M, et al. Gland Instance Segmentation by Deep Multichannel Neural Networks. arXiv preprint arXiv:160704889. 2016; p. 1–10.

[41] Ciresan D, Giusti A, Gambardella L, Schmidhuber J. Deep neural networks segment neuronal membranes in electron microscopy images. Advances in Neural Information Processing Systems. 2012; p. 2843–2851.

[42] Greenspan H, Van Ginneken B, Summers RM. Guest Editorial Deep Learning in Medical Imaging: Overview and Future Promise of an Exciting New Technique. IEEE Transactions on Medical Imaging. 2016;35(5):1153–1159. 10.1109/TMI.2016.2553401

[43] Jurrus E, Paiva ARC, Watanabe S, Anderson JR, Jones BW, Whitaker RT, et al Detection of neuron membranes in electron microscopy images using a serial neural network architecture. Medical Image Analysis. 2010;14(6):770–783. 10.1016/j.media.2010.06.002 20598935 PMC2930201

[44] Teikari P, Santos M, Poon C, Hynynen K. Deep Learning Convolutional Networks for Multiphoton Microscopy Vasculature Segmentation. arXiv preprint arXiv:160602382. 2016; p. 1–23.

